# Understanding the dispensary workflow at the Birmingham Free Clinic: a proposed framework for an informatics intervention

**DOI:** 10.1186/s12913-016-1308-7

**Published:** 2016-02-19

**Authors:** Arielle M. Fisher, Mary I. Herbert, Gerald P. Douglas

**Affiliations:** Center for Health Informatics for the Underserved, Department of Biomedical Informatics, School of Medicine, University of Pittsburgh, Pittsburgh, PA USA; Program for Health Care to Underserved Populations/Birmingham Free Clinic, Pittsburgh, PA USA

**Keywords:** Vulernable populations, Needs assessment, Qualitative research, Workflow, Electronic health records, Information systems, Pharmacy, Medical informatics applications

## Abstract

**Background:**

The Birmingham Free Clinic (BFC) in Pittsburgh, Pennsylvania, USA is a free, walk-in clinic that serves medically uninsured populations through the use of volunteer health care providers and an on-site medication dispensary. The introduction of an electronic medical record (EMR) has improved several aspects of clinic workflow. However, pharmacists’ tasks involving medication management and dispensing have become more challenging since EMR implementation due to its inability to support workflows between the medical and pharmaceutical services. To inform the design of a systematic intervention, we conducted a needs assessment study to identify workflow challenges and process inefficiencies in the dispensary.

**Methods:**

We used contextual inquiry to document the dispensary workflow and facilitate identification of critical aspects of intervention design specific to the user. Pharmacists were observed according to contextual inquiry guidelines. Graphical models were produced to aid data and process visualization. We created a list of themes describing workflow challenges and asked the pharmacists to rank them in order of significance to narrow the scope of intervention design.

**Results:**

Three pharmacists were observed at the BFC. Observer notes were documented and analyzed to produce 13 themes outlining the primary challenges pharmacists encounter during dispensation at the BFC. The dispensary workflow is labor intensive, redundant, and inefficient when integrated with the clinical service. Observations identified inefficiencies that may benefit from the introduction of informatics interventions including: medication labeling, insufficient process notification, triple documentation, and inventory control.

**Conclusions:**

We propose a system for Prescription Management and General Inventory Control (RxMAGIC). RxMAGIC is a framework designed to mitigate workflow challenges and improve the processes of medication management and inventory control. While RxMAGIC is described in the context of the BFC dispensary, we believe it will be generalizable to pharmacies in other low-resource settings, both domestically and internationally.

**Electronic supplementary material:**

The online version of this article (doi:10.1186/s12913-016-1308-7) contains supplementary material, which is available to authorized users.

## Background

Inadequate access to health care can cause health disparities in medically vulnerable populations, such as the homeless and uninsured. Chronic medical conditions are prevalent in these populations, with nearly half of all uninsured, non-elderly American adults plagued by at least one chronic illness [1]. Further, more than half of those uninsured with a chronic illness report that they did not seek required medical care or purchase prescription drugs due to cost [1]. Ensuring an uninterrupted drug supply to these patients is a critcal part of providing sufficient health care in a low-resource setting, both domesitically and internationally [2].

Therapeutics is an essential component of every health system and largely relies on accurate and timely management of the drug supply chain, which includes the procurement, shipping, storage and dispensing of medication to patients [2]. Stockouts are frequently encountered in low-resource settings, particularly in developing nations, and can have detrimental effects on patient care [2]. Accurate information on currrent stock counts and projections of future medication requirements is necessary to minimize stockouts and expired stock, keeping critical drugs on the shelves and preventing low-usage medications from expiring. This task reqiures pharmacists with appropriate skills and management tools [3], as unreliable monitoring of medication utilization is a contributing factor to supply chain problems in low-resource settings [4]. The effective provision of these services through safety net providers is necessary to decrease hospitalizations, Emergency Department visits, and associated health care costs in medically vulnerable populations [5]. In this paper, we present supply chain challenges in the case of a safety net clinic in Pittsburgh, Pennsylvania, USA, and how inefficiencies in workflow processes can contribute to these challenges.

In response to seeing medically vulnerable individuals discharged from hospitals without follow-up care, the Program for Health Care to Underserved Populations (PHCUP) was founded in 1994 under the sponsorship of the University of Pittsburgh Medical Center (UPMC) [6]. The PHCUP partnered with The Salvation Army in Pittsburgh, PA, USA to create the Birmingham Free Clinic (BFC) in an effort to reduce health disparities by providing quality health care, pharmaceutical, and social services to uninsured and vulnerable individuals. The BFC is the Program’s longest-running initiative and is currently the only free, non-federally-funded, walk-in health clinic in the city of Pittsburgh, providing clinical services to approximately 1,900 patients annually [6]. This volunteer-staffed clinic addresses the diverse needs of the uninsured, homeless, transitionally housed, and working class communities of this city. The BFC provides a wide range of clinical services, including the identification and prevention of disease, management of chronic conditions, and referrals for health and social services. Additionally, the BFC provides on-site access to essential medications for its patients through the use of an in-house dispensary managed by one to two volunteer pharmacists during a given clinic session. Pharmacists also assist patients in applying for Patient Assistance Programs (PAPs), offered voluntarily by most pharmaceutical manufacturing companies, which provide prescription drugs to low-income persons who lack drug coverage.

Medication Therapy Management (MTM) and disease state management services are essential at the BFC, as the dispensary provides on average 2–3 prescriptions per patient which results in nearly 8,400 prescriptions dispensed annually. MTM is a service promoting safe and effective medication use through patient-pharmacist-prescriber parternships, in which pharmacists identify, mitigate, and resolve medication-related problems [7], and has proven to be effective in underserved settings [8]. Between 300 and 400 BFC patients are enrolled in PAPs and undergoing monthly MTM which is nearly 20 % of the total patient population. The pharmacists at the BFC dispensary have implemented a systematic, paper-based approach to delivering these services to their patients. However, inefficiencies regarding this labor-intensive system require the pharmacists to spend a significant amount of time documenting patient visits as opposed to providing patient care. In an effort to standardize clinical care practices and eliminate paper-based reporting at the BFC, an electronic medical record (EMR) was implemented in the clinic in 2013.

The American Medical Association states that meaningful use of an EMR can improve the patient experience (including quality and satisfaction), improve the health of populations, and reduce the per capita cost of health care [9]. These benefits cannot be fully realized in the BFC dispensary due to the inability of the EMR to support workflow between the clinical service and the dispensary. The introduction of the EMR has improved several aspects of the overall clinic workflow, such as the ability to electronically review past medications, outpatient visits, and laboratory results for any BFC patient seen at any UPMC outpatient clinic or hospital. However, the EMR only addresses a portion of the clinic services while failing to integrate with the dispensary workflow, making communication between both entities more challenging. Many of the pharmacists report that the EMR has not directly improved their efficiency, creating redundancy in many of their tasks.

While many of the clinic operations have become automated through the introduction of the EMR, the dispensary continues to maintain a paper-based dispensing process. The BFC dispensary also lacks a complete inventory control system, which can have detrimental effects in the case of a medication stock-out. While the BFC has not experienced many medication stock-outs to date, the inventory reporting system is laborious, inefficient, and only managed by one person. Inefficiencies in the BFC dispensary workflow may have existed prior to EMR implementation, but the move to an EMR has exacerbated these inefficiencies by creating a boundary between the medical and pharmaceutical services. The medication management process is burdened by the workflow challenges associated with the EMR such as redundant documentation tasks.

We aim to provide informatics interventions to these challenges guided by a detailed understanding of the problems and information needs of the primary user: pharmacists. However, identifying workflow inefficiencies and designing interventions to alleviate them is a complex task. Many qualitative methods, such as interviews, focus sessions, and surveys, can be used to collect feedback from users to identify requirements for designing a new system. While these methods can be useful, they depend on the user’s ability to clearly articulate his/her needs, which can be a difficult task. Other methods, such as task analysis, provide a comprehensive framework to understand what the users’ goals are and how they go about ahcieving those goals. However, this process specifically focuses on the user group of interest, while not consiering how this group interacts with other cultural entities and physical artifacts.

Holtzblatt et al. designed contextual inquiry as a user-centered, social method aimed to identify and understand users’ needs and unarticulated knowledge about work [10]. This methodlogy utilizes direct work observation and four graphical models to describe all aspects of workflow, both phsycial and cultural, and provides highly-detailed data about the structure of work practice needed to design products [10]. Several studies have demonstrated the effective use of contextual inquiry to uncover workflow inefficiencies in clinical settings and to inform the design of medical devices and information systems [11–13]. Therefore, we utilized contextual inquiry in this needs assessment study to identify workflow challenges in the BFC dispensary. In this paper we present these challenges, as perceived by the pharmacists, and propose a framework for an informatics intervention to alleviate them.

## Methods

**Fig. 1:**
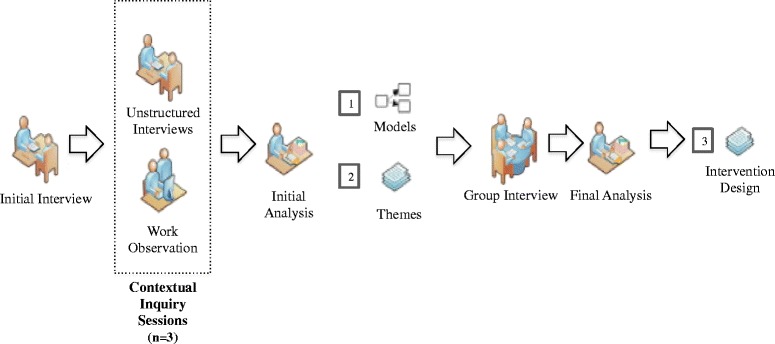
Methodology overview. Graphical overview of the study design. Figure adapted from Turner et al. [14]

To investigate the information needs of the key users and provide formal documentation of their workflow, we conducted contextual inquiry sessions with the BFC pharmacists during clinical care sessions according to the guidelines suggested by Holtzlblatt et al. [10]. Contextual inquiry sessions included both direct work observation and informal interviews, consisting of unstructured questions about certain things the user was doing during observation. We used data from the contextual inquiries to create four graphical models that were validated during a group discussion with the selected pharmacists. We then completed open coding and categorization of the observer notes to identify a set of themes summarizing the primary challenges and inefficiencies encountered by the pharmacists at the BFC dispensary. To focus our intervention design on the five highest-ranking challenges, we asked the pharmacists to rank the themes in order of their significance. Based on our findings, we proposed a framework for an informatics intervention designed to alleviate the workflow challenges. Figure 1, adapted from a study by Turner et al. [14], provides an overview of our methodology.

### Contextual inquiries

Three pharmacists who regularly volunteer at the BFC dispensary were observed while completing routine dispensing tasks during general clinic hours. These tasks included, for example, medication dispensing, patient counseling, prescription documentation, and inventory maintenance. All three pharmacists have received their Doctor of Pharmacy and are affiliated with the University of Pittsburgh School of Pharmacy. Additionally, all of the selected pharmacists have experience providing care in underserved settings. The University of Pittsburgh Institutional Review Board approved this study as “exempt” and considered it to be quality improvement research (PRO14020120). No personally identifiable patient or pharmacist information was collected or recorded, and there was no interaction between patients and researchers; thus informed consent was not required from participants. On behalf of the BFC, the medical director offered her written consent and support of this study.

Three contextual inquiry sessions were conducted at the BFC dispensary. The number of dispensed prescriptions and patients encountered at each session varied according to the specialty clinic type, available services, patient volume, and pharmacist. Each session lasted approximately three to four hours, followed by a brief discussion with the pharmacists to discuss the observer’s interpretation of the data. Two additional sessions were conducted to eliminate any residual confusion around data interpretation; these sessions lasted approximately three hours each.

A single researcher (AF) conducted the contextual inquiry sessions. During each session, the researcher utilized the master-apprentice model by frequently asking questions of the pharmacist (the “master”) while he or she dispensed medications and interacted with patients [10]. Notes representing user-provided data such as key user needs, information sources and flow, physical artifacts, activities, and regulatory tasks were captured during contextual inquiries. The researcher also noted the occurrence of “breakdowns” that are defined as anything or anyone that interrupted the pharmacist and/or kept the pharmacist from completing a given task [12]. In addition to breakdowns, we also documented successful aspects of the current dispensary workflow so that we can take them into consideration during intervention design.

### Data interpretation

After the conclusion of the final contextual inquiry session, we produced four graphical models to facilitate data visualization and understanding: physical model, artifact model, sequence model, and cultural model. The physical model captured the actual layout of the BFC to portray different components of the environment that may support or hinder the pharmacist’s work. We created this model using photographs and sketches produced during contextual inquiry sessions. The artifact model documented and described any physical objects (i.e., medication labels, forms) or devices that are necessary to understand the structure of the dispensary workflow. The sequence model captured the main steps pharmacists take to perform their work tasks and described how workflow strategies differ between users. The cultural model illustrated the expectations, goals, values, and general policies that may influence how the pharmacists accomplish their work and coordinate information flow with co-workers [10].

We analyzed the written results from a single contextual inquiry session within 48 h of each session to identify frequently occurring breakdowns. After the final contextual inquiry session, we analyzed all notes and graphical models collected from the three sessions. Many of the breakdowns and workflow inefficiencies documented during observations fell into distinct work tasks which were categorized. These categories informed a consolidated list of themes describing the most common workflow inefficiencies and challenges encountered by pharmacists in the dispensary.

### Member checking

We conducted a member check with the pharmacists to validate our interpretation and analysis of their workflow challenges using the graphical models. Member checking is a quality control process used to improve the accuracy and validity of what has been collected during inquiry sessions. This discussion lasted one hour and was audio-recorded. We asked the pharmacists to individually rank the list of themes by level of importance to focus our intervention designs to the five most relevant workflow challenges. We applied a weighted ranking technique to produce a new, harmonized list of workflow challenges.

To assess the degree to which the pharmacists agreed on their rankings, we computed two Kendall tau rank correlation coefficients using RStudio [15]. This statistic measures the association between separate entities (pharmacists) when both rank the same data [16]. The first coefficient measured the correlation for the entire ranked list while the second coefficient measured the correlation between the top 5 challenges in each list.

## Results

Three pharmacists participated in this study. Table 1 describes their characteristics, including how often they volunteer at the BFC.

**Table 1:** Characteristics of pharmacists

Pharmacist	Position	Clinic (hours/week)	Time at BFC (years)
A	Pharmacy resident (Global health/Underserved track)	4	2
B	School of Pharmacy faculty	12	7
C	School of Pharmacy faculty	4	10

### Data interpretation

**Fig. 2:**
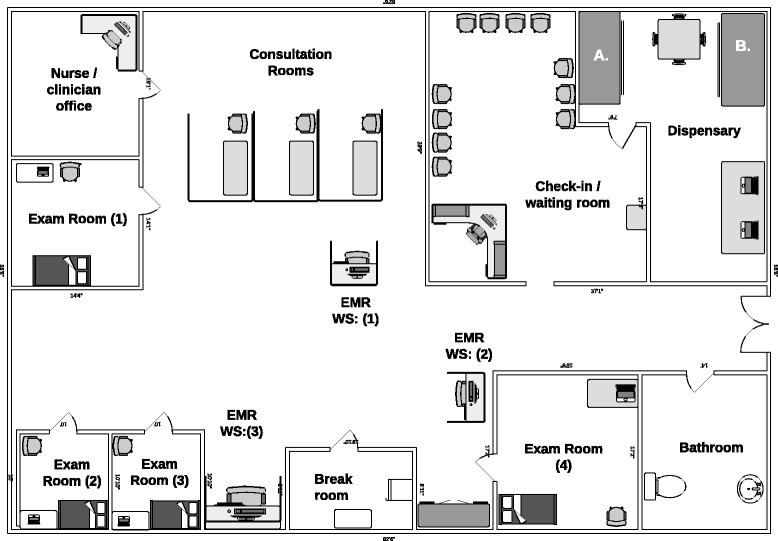
Birmingham Free Clinic floor plan. EMR = electronic medical record, WS = workstation, **A** = PAP medication storage cabinet, **B** = General medication storage cabinet

Figure 2 illustrates the floor plan of the BFC, including the pharmacists’ working area. The dispensary occupies a single room approximately 150 sq. ft. in area and contains a desk for two computers with EMR access, a table for patient counseling, and two separate medication cabinets: PAP and the general stock. Each examination room also includes a laptop with EMR access.

We collected two physical artifacts from the BFC: medication labels and the Pharmacy Activity Sheet. For every medication that is dispensed, pharmacists handwrite medication labels and tape them onto bottles. An average of 25 medication labels are handwritten and affixed to bottles each clinic session, with many of these same patients returning to the clinic each month for a prescription refill. Medication labels are a three-part form with two carbon copies that are each used for different purposes such as past dispensation records. Similar dispensation and important inventory information is then written on the Pharmacy Activity Sheet which is sent to the PHCUP offices at UPMC Montefiore Hospital in Pittsburgh, PA. Pharmacists use this form to indicate PAP medication dosage changes or low inventory medications. It is important to note that these procedures, such as the Pharmacy Activity Sheet, fulfill certain FDA rules and regulations regarding free clinics and dispensary management that require the pharmacists to complete these processes. Further description and illustration of these artifacts can be found in Additional files 1 and 2.

To understand the flow of these artifacts and other information throughout the clinic, we define five main activities that all pharmacists complete to dispense medication. The overall events that contribute to the receipt and dispensation of medications at the clinic are described below:

A. Medication retrieval
  Volunteers document the receipt of PAP medications arriving at the PHCUP office and update the status of a patient’s PAP application. A ‘Date to Re-Order’ label if affixed to each PAP medication bottle so that the pharmacists remember to document this re-order on the Pharmacy Activity Sheet. PAP medications are transferred to the BFC from the PHCUP office and are stocked in a specific cabinet with patient name labels to ensure the correct patient is receiving the medication.
B. Prescription preparation
  When a clinician decides to order medications for a patient, he or she does so electronically in the EMR. The clinician then must update the patient status icon on the EMR dashboard to signify that the patient requires pharmacy services. Upon noticing the status change, pharmacists review prescription information and check medication stocks to ensure the dispensary can fill the prescription. If the dispensary does not have the medication, the pharmacist must communicate this to the clinician so they can devise a new treatment plan. This often creates extra work for both the pharmacist and the clinician; the pharmacist is typically inclined to interrupt the clinician while he or she is examining the next patient.
C. Dispensation
  The pharmacist consults the cabinets for the medication stock. The correct number of pills are counted and filtered into a patient-supplied medication bottle or a new medication bottle supplied by the dispensary. The medication labels are written and affixed to medication bottles.
D. Patient counseling
  Pharmacists counsel each patient individually during dispensation which is a critical aspect of medication management in an underserved setting. The pharmacists adopt a ‘show-and-tell’ style to explain and illustrate medication usage to patients.
E. Pharmacy Activity Sheet
  Pharmacists fill out the Pharmacy Activity Sheet following the last patient counseling session. To complete this document, they typically revisit each patient record in the EMR to acquire the prescription information. Additionally, the pharmacists must remember inventory information that can be a cognitive burden at the end of a 4-h clinic session.

To assess the different strategies that pharmacists employ when completing these tasks, we used a sequence model to capture the sequence of steps and breakdowns that may occur during each activity. The consolidated sequence model (Additional file 3) combines findings from six individual sequence models. We found that pharmacists encounter the most breakdowns and additional steps when they need to discuss a new medication plan with a clinician. This typically occurs when a clinician orders a medication or dose that the dispensary does not have. Breakdowns during this process were attributed to the complexity of correcting an incorrect order in the EMR and, primarily, the lack of visibility clinicians have into the current inventory. Due to the high-turnover rate of resident and intern volunteers, in addition to a limited formulary, providers are often unaware what the pharmacists are able to dispense. This proved to be one of the most frequent and frustrating breakdowns that we documented during observations. The occurrence of this breakdown results in the creation of several required, but inefficient, tasks. For example, these include a cumbersome EMR correcting process, discussing a new medication plan with a clinician, and potentially interrupting a clinician while he or she is consulting another patient.

**Fig. 3:**
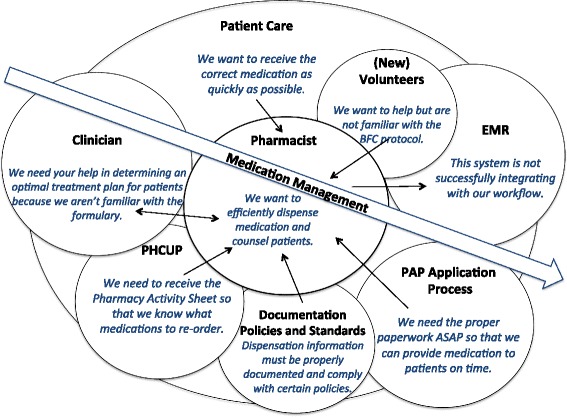
Cultural model. The central arrow represents the main goal of the pharmacists (medication management). The overlapping circles show the main influencs on the pharmacists; size of the circle indicates degree of influence. Text in italics describes the primary concerns of each influencing factor

The cultural model (Fig. 3) illustrates the degree to which different organizations, general policies, values, and relationships influence the dispensary workflow. The most influential process, medication management, is represented by the central arrow. Incorrect order entries in the EMR create unnecessary work for the pharmacists and significantly influence their dispensation inefficiency. Additionally, documentation tasks are time-consuming and redundant which sometimes results in errors and miscommunication between the clinic dispensary and the PHCUP administrating office at UPMC Montefiore.

### Workflow challenge themes

The graphical models facilitated identification of 13 categories that describe workflow challenges in the dispensary. Table 2 displays these categories and their problem definitions in their order of importance as perceived by the pharmacists. We provide direct pharmacist quotations collected during observation sessions to further explain each theme in Additional file 4.

**Table 2:** Workflow challenge themes

**Theme**	**Definition**
**Labeling**	**The labeling of medication bottles is time-consuming, redundant, incomplete, and can lead to inaccuracies.**
**Insufficient Process Notification**	**The EMR has an inefficient method of alerting the pharmacist that a patient is ready for dispensary services due its reliance on the clinician’s memory.**
**Triple Documentation**	**The pharmacists currently document patient prescription/dispensation information in three separate documentation forms (i.e., medication labels, Pharmacy Activity Sheet, and the EMR).**
**Knowledge of Patient Details During CPOE**	**Clinicians are unaware of a patient’s financial capabilities during CPOE, that determine what type of medication and dosage to be ordered.**
**Clinician Ordering**	**The destination of the patient prescription (i.e., BFC, community pharmacy) is unknown during CPOE, which often leads to incorrect CPOE.**
Knowledge of Formulary	Clinicians have no visibility into the available medication stock at the BFC dispensary.
Dispensing	The EMR is only capable of supporting the prescribing process at the BFC and does not provide a similar structure for dispensing practices.
Patient Validation	The dispensary lacks an explicit method to validate that the right medication is being dispensed to the right patient.
Inventory Maintenance	The dispensary is unable to use prescription information in EMR to track medication inventory and dispensing history in real-time.
Drug-Drug Interactions (DDI)	The dispensary lacks an explicit method for checking potential DDI’s at the counseling site due to the inability to readily access past dispensation records.
EMR Accessibility	Pharmacists cannot readily access the EMR at relevant locations, such as the patient counseling location.
EMR Complexity	Volunteer pharmacists and clinicians are not familiar with the specific protocol when ordering medications to the BFC dispensary in the EMR which leads to incorrect EMR order entries.
PAP Application Process	The PAP application process is challenging and time consuming due to the large amount of paperwork involved and the wide variation in application format and patient requirements.

Items in bold are the highest ranked workflow challenges as perceived by the pharmacists. CPOE = Computerized phsyician order entry

### Assessment of judges

We computed three pairwise Kendall tau rank correlation coefficients between pharmacists (A, B, and C) in Table 3. These values demonstrated little similarity between the pharmacists’ ranked lists. We attributed these results to differences in pharmacist experience at the BFC. Pharmacist A began volunteering at the BFC after the EMR was deployed, thus having little understanding of the workflow before this systematic change. Pharmacists B and C have legacy perceptions as they experienced this change in workflow and likely have a better understanding of how the EMR exacerbated certain inefficiencies. Because we were primarily interested in the highest-ranking inefficinecies, we also computed three coefficients between the top five in each of the pharmacists’ lists (Table 3). The coefficients for this comparison were much stronger, although pharmacists A and C still lacked any similarity. In an informal discussion, all pharmacists verbalized their overall support for the list, giving us motivation to proceed with proposing interventions.

**Table 3:** Kendall tau correlation coefficients

Pairwise tests	τ (entire lists)	τ (top 5 rated themes)
A and B	0.03	0.40
A and C	0.00	0.00
B and C	0.31	0.60

## Discussion

We believe this is the first study using contextual inquiry to model dispensary workflow and uncover process challenges and breakdowns in a low-resource setting. While the BFC dispensary workflow has undergone many refinements since its inception, we noted much inefficiency and redundancy in the dispensing process. In this study, we identified 5 themes describing workflow challenges: labeling, insufficient process notification, triple documentation, knowledge of patient during computerized physician order entry (CPOE), and clinician ordering. The pharmacists perceived redundancy in the latter two themes, thus, we coalesced these ideas into one theme (CPOE) to reduce ambiguity. These workflow challenges were uncovered at the BFC dispensary, but we believe pharmacists may encounter similar challenges in any low-resource clinic. Thus, designing an informatics intervention to alleviate these challenges may be useful in pharmacies with similar workflow challenges.

To our knowledge, limited research exists on the challenges pharmacists encounter when serving an under-resourced population. This research primarily focuses on the challenging use of Patient Assistance Programs in safety-net clinics and low-resource pharmacy settings. Research done by Duke et al. concluded that, while these programs fill a major gap in health insurance coverage, they consume nearly 12 h of pharmacist time per month [17]. Further, they stated that standardized application procedures are needed to closely manage this process [17]. Software tools exist to manage the PAP application process, but they have been shown to be quite expensive and unable to meet the needs of every organization [17, 18]. This software enhances the PAP application process but fails to include additional pharmacy functionality, such as streamlined dispensing and inventory management. A low-resource setting, such as the BFC, does not have funds available to contribute to a specific software program that will not support all aspects of their workflow. Rather than implementing several, modular interventions, i.e., PAP software, we believe that the introduction of an information system at the dispensary level will provide a more standardized, systematic approach to the dispensing and PAP application process.

We propose a resource for Prescription Management and General Inventory Control (RxMAGIC) that is designed to improve the process of medication management. We intend for RxMAGIC to be an integrated information system grounded in several informatics interventions. The proposed resource will be an open-source, stand-alone system with the ability to interoperate with the current EMR system at the clinic. RxMAGIC will have 3 high-level functions that are designed to alleviate the specific workflow challenges identified in this study: inventory control, streamlined dispensation, and PAP application management. We describe three primary functional components of RxMAGIC in the context of the highest-ranking workflow challenges, as identified below:

1. Computerized physician order entry
  Lack of visibility into the current medication inventory results in most challenges associated with CPOE. Clinicians frequently consult the pharmacists during patient examinations regarding the availability of certain medications. Confusion around the inventory often leads to incorrect EMR order entries which creates extra work for the pharmacists during clinic hours and can result in unreliable monitoring of medication utilization. The proposed framework will help the pharmacists manage stock levels in the dispensary by keeping track of each dispensed unit, automatically adjusting the inventory as medication is dispensed, and alerting the pharmacists of low medication inventory. This may yield more accurate stock estimates which can inform future medication orders. A web-based inventory system has proven to be successful in avoiding drug stockouts in other low-resource settings [4, 19]. The web interface will give clinicians visibility into the medication inventory while simultaneously viewing the EMR on the same computer. This will create a parallel representation of the patient profile, including unique attributes such as patient income that can be concurrently viewed by physicians and pharmacists.
2. Labeling and triple documentation
  Labeling medication bottles is a routine task that requires significant time and attention to detail. As the volume of patient prescriptions increases, the labeling process becomes more burdensome; the process has poor scalability. RxMAGIC is designed to streamline the dispensing process by producing computer-generated medication labels with unique barcodes to tightly couple the dispensed unit to the receiving patient. Thus, each computer in the dispensary will have an attached barcode scanner and thermal label printer to efficiently produce medication labels [20]. This scalable method will also maintain past dispensation records for each patient that will replace the current paper-based filing system used at the free clinic. RxMAGIC will utilize electronic prescription information to automatically generate reports with patient dispensation data which is intended to alleviate redundant documentation.
3. Insufficient process notification
  The process for notifying pharmacists that a prescription is ready relies on the clinician to update the “patient dashboard” in the EMR and for the pharmacists to notice this change, which often results in unnecessary delays. In an effort to respect a patient’s time constraints, pharmacists may only have little time to counsel patients. Minimal education regarding medication therapy is problematic in medically vulnerable populations [21]. RxMAGIC will automatically alert pharmacists that a clinician has placed a medication order in the CPOE which will give pharmacists more time to prepare the prescription and counsel the patient. We will also implement a visible dashboard displaying patient information and their associated status in the clinic (e.g., clinician examination, ready for pharmacy services). To enhance the PAP application process, we also propose that RxMAGIC alert the pharmacists of approaching PAP re-enrollment and medication re-order dates to avoid patient-specific stockouts.

### Limitations

We recognize that contextual inquiries by a single researcher is a limitation of this study. However, the pharmacists were actively involved in validating and modifying the workflow themes. We believe this user validation will significantly improve the design of RxMAGIC. While the Kendall tau coefficients were quite low for the entire ranked list, signifying little similarity between the individually ranked lists, the coefficients for the highest-ranking challenges were somewhat higher. This demonstrated greater agreement among the top five challenges in each individually ranked list, which was the focus of our intervention design. The pharmacists reported that ranking the themes was quite difficult, as they were not able to express the magnitude of importance of each theme in relation to other themes. To this end, we plan to complete a time-motion study to quantitatively assess the impact of each workflow challenge. This experiment will allow us to measure how much time the pharmacists spend on various tasks which will act as a triangulation component to this study.

## Conclusions

Our contextual inquiry uncovered inefficient aspects of the dispensary workflow that may be alleviated through introduction of an informatics intervention. We described RxMAGIC, a framework to mitigate workflow challenges and streamline the dispensing process. While RxMAGIC was defined in the context of the BFC dispensary, we believe that its functionality may be generalizable to pharmacies in other low-resource settings, both domestically and internationally. In future research, we will design, build, and deploy RxMAGIC and evaluate its impact. We expect to improve pharmacist efficiency so they can spend more time providing quality patient care and less time documenting patient visits in redundancy. Visibility of the inventory will allow clinicians to have more informed conversations with patients during examinations, so they can determine an optimal treatment plan in a timely fashion. We expect that the proposed informatics system will improve important aspects of the medication management process which is critical to delivering care in a medically vulnerable population.

## Additional files

Additional file 1:
**Mock medication label.** Physical artifact model describing the use of medication labels in the BFC dispensary. PAP = Patient Asssistance Program. (PDF 91 kb) Additional file 2:
**Pharmacy activity sheet.** Physical artifact model describing the use of the pharmacy activity sheet in the BFC dispesnary. (PDF 243 kb) Additional file 3:
**Sequence model.** This model lists the ordered steps required to complete various activities in the dispensary. The breakdowns that may occur at each step are also listed. *Abstract strategy* represents a distinct pharmacist workflow that deviates from the typical workflow. PAP = Patient Assistance Program; EMR = Electronic medical record. (PDF 84 kb) Additional file 4:
**Pharmacist quotations to explain workflow challenge themes.** Items in bold are highest ranked workflow challenges as perceived by the pharmacists. (PDF 63 kb)

## Abbreviations

BFC: Birmingham Free Clinic
CPOE: computerized physician order entry
DDI: drug-drug interaction.
EMR: electronic medical record
MTM: Medication Therapy Management
PAP: Patient Assistance Program
PHCUP: Program for Health Care to Underserved Populations
RxMAGIC: Prescription Management and General Inventory Control
UPMC: University of Pittsburgh Medical Center
