# Supplementary material for: Understanding the dispensary workflow at the Birmingham Free Clinic: a proposed framework for an informatics intervention
Source: BMC Health Serv Res. 2016 Feb 19;16:69. doi: 10.1186/s12913-016-1308-7 (PMC4759722; doi:10.1186/s12913-016-1308-7)
Supplement: Additional file 1: — Mock medication label. Physical artifact model describing the use of medication labels in the BFC dispensary. PAP = Patient Asssistance Program. (PDF 91 kb) [file 12913_2016_1308_MOESM1_ESM.pdf]

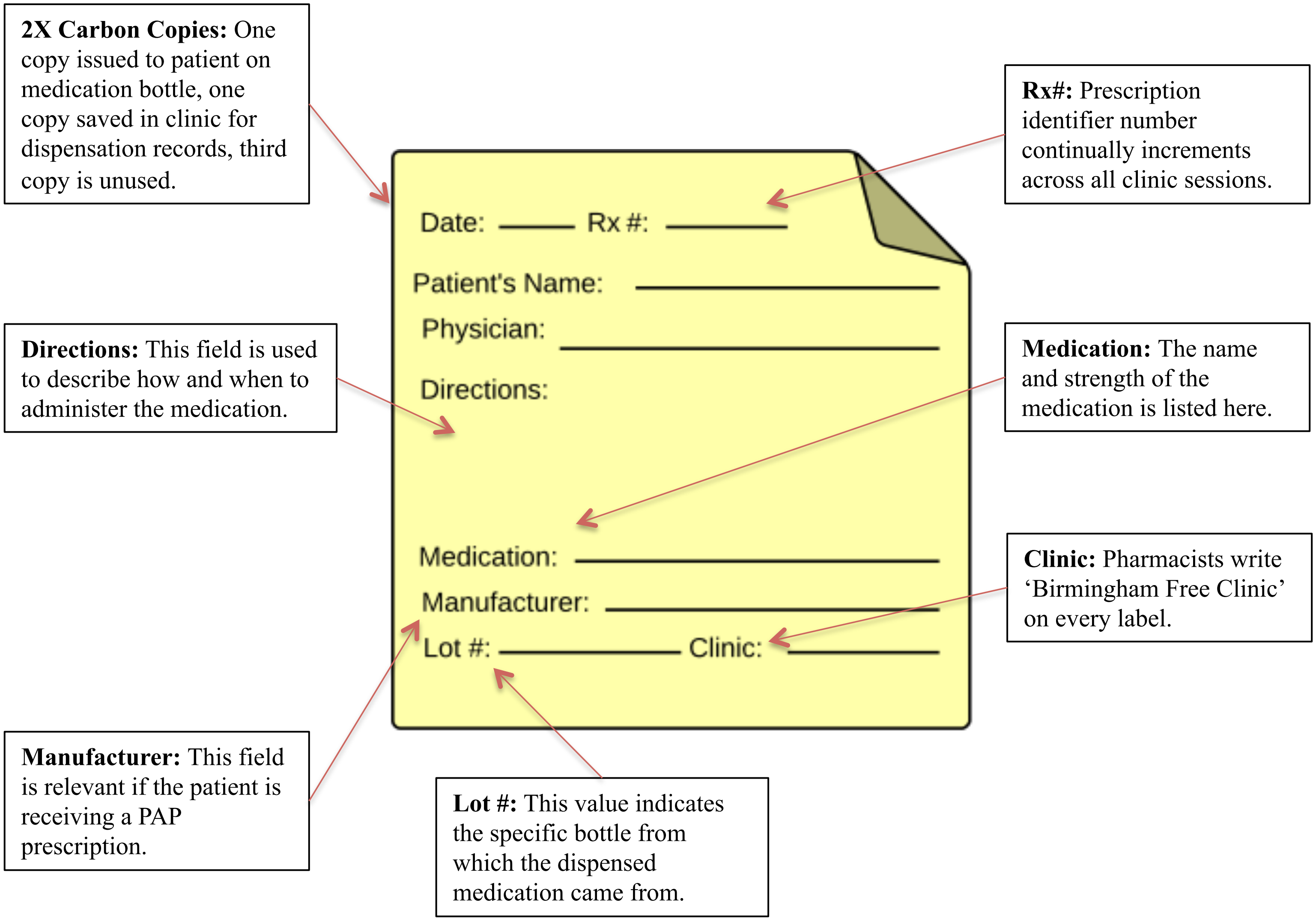

2X Carbon Copies: One copy issued to patient on medication bottle, one copy saved in clinic for dispensation records, third copy is unused.
Date:
Rx #:
Patient's Name:
Physician:
Directions:
Medication:
Manufacturer:
Lot #:
Clinic:
Rx#: Prescription identifier number continually increments across all clinic sessions.
Directions: This field is used to describe how and when to administer the medication.
Medication: The name and strength of the medication is listed here.
Clinic: Pharmacists write 'Birmingham Free Clinic' on every label.
Manufacturer: This field is relevant if the patient is receiving a PAP prescription.
Lot #: This value indicates the specific bottle from which the dispensed medication came from.
