# Supplementary material for: Understanding the dispensary workflow at the Birmingham Free Clinic: a proposed framework for an informatics intervention
Source: BMC Health Serv Res. 2016 Feb 19;16:69. doi: 10.1186/s12913-016-1308-7 (PMC4759722; doi:10.1186/s12913-016-1308-7)
Supplement: Additional file 2: — Pharmacy activity sheet. Physical artifact model describing the use of the pharmacy activity sheet in the BFC dispesnary. (PDF 243 kb) [file 12913_2016_1308_MOESM2_ESM.pdf]

Pharmacy Activity Sheet

Source of Today's Meds: This field is used to indicate the specific stock the dispensed medication came from; it is used for inventory purposes.

Date ______________________ Pharmacist ______________________

Clinic ______________________ Student ______________________

Please fill out all columns for each patient with only one medication per line.

Patient Name	Med & #Dispensed	Sig.		Source of Today's Meds				PMAP MED ORDERS				Comments: (App started/Pending App/Name of Pt Med Borrowed From, Income doc status, etc.)
				Borrowed	Sample	Stock	PMAP	New Med	Re-Order	Dose Change	New App or Re-Enroll	
Ex: Jane Doe	Protonix 40mg #30	T BID	AT			✓		✓			N / RE	Pfizer application started for patient, will fax income docs
											N / RE	
											N / RE	
											N / RE	
											N / RE	
											N / RE	
											N / RE	
											N / RE	
											N / RE	

PMAP Med Orders: Pharmacists specify if a PAP medication needs to be re-ordered in this field. Also, pharmacists use these boxes to request a dose change.

Stock meds to be replenished:

1. ______________________
2. ______________________
3. ______________________
4. ______________________
5. ______________________
6. ______________________

NOTES:

Stock meds.: This section of the document is to report low inventory for 'General' medications.

Comments: This box is used to communicate the status of a PAP application to the offices at UPMC Montefiore.
