# Supplementary material for: Understanding the dispensary workflow at the Birmingham Free Clinic: a proposed framework for an informatics intervention
Source: BMC Health Serv Res. 2016 Feb 19;16:69. doi: 10.1186/s12913-016-1308-7 (PMC4759722; doi:10.1186/s12913-016-1308-7)
Supplement: Additional file 3: — Sequence model. This model lists the ordered steps required to complete various activities in the dispensary. The breakdowns that may occur at each step are also listed. Abstract strategy represents a distinct pharmacist workflow that deviates from the typical workflow. PAP = Patient Assistance Program; EMR = Electronic medical record. (PDF 84 kb) [file 12913_2016_1308_MOESM3_ESM.pdf]

**Activity**<br>***Intent/Trigger/Abstract Strategy***	**Tasks**	**Breakdowns**
Intent: **Prepare 'General' prescription to be dispensed.**<br><br>***Trigger:*** **Clinician places a medication order (non PAP).**<br><br>***Abstract Strategy:*** To expedite the process, pharmacist prepares medication orders for patients with chronic conditions prior to clinician examination.	**T1.** Scan EMR dashboard to see if patient status has changed to a blue icon.<br>**T2.** Receives prescription information in EMR.<br>**T3**. Scans stock cabinets for medication.	**B1**. Clinician failed to update patient status after examination.<br>**B2**. The dispensary does not have the prescribed medication.
Intent: **Prepare PAP medications to be dispensed.**<br><br>***Trigger:*** **An PAP patient has checked in at the clinic and has met with the pharmacist.**	**T1**. If patient is a monthly scheduled patient, then pharmacist has a one-on-one counseling session with the patient in an exam room.<br>**T2.** Medications are retrieved from PAP cabinet.<br>**T3.** Medication labels are prepared and taped on top of previous labels.<br>**T4.** Medications are dispensed immediately to patient (patient only requires clinician examination every three visits).	**B3**. PAP medications have not been re-ordered.<br>**B4.** Patient was due for an annual program re-enrollment but paperwork has not been processed.
Intent: **Discuss new medication plan with clinician.**<br><br>***Trigger:*** **Clinician prescribes a medication that the dispensary does not have.**<br><br>***Abstract Strategy:*** Pharmacist feels comfortable determining a new treatment plan for the patient without consulting the clinician.	**T1.** Find clinician in the clinic during clinic session.<br>**T2.** Consult clinician to discuss the problem.<br>**T3**. Determine new treatment plan with clinician.<br>**T4**. Scan medication cabinets for new medication.<br>**T5**. Correct EMR entry to adjust for the new order.	**B5**. Pharmacist must leave his/her workspace to interrupt a clinician while he/she is with a new patient.<br>**B6**. Clinician does not remember all of the details from a recent patient and must refer to EMR.<br>**B7**. The prescribed medication is required; thus, patient must buy the medication from community pharmacy.<br>**B8.** EMR order correction process is cumbersome and time-consuming.

Intent: **Dispense medications.**<br><br>***Trigger:*** **Patient prescriptions are prepared and ready to be filled.**	**T1**. All medication stock bottles are located and retrieved from cabinets.<br>**T2**. New patient-medication bottles are retrieved from drawers.<br>**T3.** Pharmacist counts the amount of pills to be dispensed.<br>**T4**. Pills are filtered into new medication bottles.<br>**T5**. Medication labels are written and affixed to bottles.	**B9**. There are not enough pills in the current stock bottle.<br>**B10**. The pharmacist miscounted and needs to start over.<br>**B11**. There is an error transcribed on the medication label and it must be re-written.<br>**B12**. Wrong medication label is taped on the medication bottle.
Intent: **PAP application initiation.**<br><br>***Trigger:*** **A low-income patient qualifies for prescription medication from a pharmaceutical manufacturing company.**	**T1**. Pharmacist explains the PAP application process to the patient.<br>**T1**. Pharmacist locates correct PAP application.<br>**T2**. Patient fills out required fields.<br>**T3**. Pharmacist and patient discuss what materials the patient must bring in to receive the medication (i.e. W2 form).<br>**T4**. Application is placed in a bin to be processed.	**B13**. There are no copies of the correct PAP application.<br>**B14**. Patient is unable to provide income documents.
Intent: **Complete the Pharmacy Activity Sheet.**<br><br>***Trigger:*** **The last patient has received medications.**	**T1**. Pharmacist retrieves new document from a binder.<br>**T2**. Pharmacist transcribes dispensed medication information from that clinic session to the form.<br>**T3**. Transcribes low inventory alerts to the proper section of the form.<br>**T4.** Deposits form in specified outgoing bin located in the check-in room.	**B15**. There are no copies of the Pharmacy Activity Sheet.<br>**B16**. Pharmacist forgets to transcribe low-inventory medication.<br>**B17.** There is insufficient time to fill out the form in its entirety.
