# Supplementary material for: Understanding the dispensary workflow at the Birmingham Free Clinic: a proposed framework for an informatics intervention
Source: BMC Health Serv Res. 2016 Feb 19;16:69. doi: 10.1186/s12913-016-1308-7 (PMC4759722; doi:10.1186/s12913-016-1308-7)
Supplement: Additional file 4: — Pharmacist quotations to explain workflow challenge themes. Items in bold are highest ranked workflow challenges as perceived by the pharmacists. (PDF 63 kb) [file 12913_2016_1308_MOESM4_ESM.pdf]

Theme	Pharmacist Quotation
**Labeling**	**"I wrote the wrong patient name on these labels, would someone fix them for me while I run back to talk to the doctor, I just don't have time."**
**Insufficient Process Notification**	**"If you're in someone's chart, you don't have the [daily] schedule open, so you don't know if a patient is ready for their prescription, and that's assuming the clinician changed the status."**
**Triple Documentation**	**"We document the same information on the label, the activity sheet, and the EMR, for every prescription we fill and for every month they come in. It's tiresome and we make mistakes."**
**Knowledge of Patient Details During CPOE**	**"I was out on the floor and saw the doctor sending a prescription to a pharmacy for 102 tablets, I asked her if we could change it to 90 tablets because I know they [community pharmacy] charge $10 for 90 tablets, and that's all that particular patient can afford. I'm glad I saw this before she sent the order!"**
**Clinician Ordering**	**"Before [EMR] it was really clear what is to be filled at the clinic versus what is sent as a prescription to another pharmacy. Now that decision is made at the point of ordering, so that makes it confusing for the doctor."**

Knowledge of Formulary	“The formularies are so different between here and what someone can get at a community pharmacy, and just because we have the medication here one time doesn’t always mean that we’ll have it here, which leads to another conversation between us, the prescriber and the patient and probably incorrect EMR orders.”
Dispensing	“The EMR only does so much for us [pharmacists], all of our dispensing practices are still the same as they were before, it has made them more time-consuming and confusing, if anything.”
Patient Validation	“It’s difficult because sometimes the patient is unsure of what he is supposed to be taking when we counsel him, so it’s important we find a way to double check that we are giving him the right medication.”
Inventory Maintenance	“I was back there [inventory cupboard] digging for Celexa, hoping there would be something there to fill this patient’s prescription, and I couldn’t find anything. It’s frustrating, if there was a system that could help…”
Drug-Drug Interactions (DDIs)	“Unless I am personally entering the medication order, I won’t see the DDI provided by the EMR. It would be great if we had some sort of system that would double check for DDIs as we were counseling the patient.”

EMR Accessibility	“Especially during the smoking cessation clinic, we have no way to access the patient’s chart during counseling to see what medications they are on.”
EMR Complexity	“The protocol of using the EMR here is tricky, because it’s not like normal care facilities, physicians use it [EMR] at their own practice, but the workflow at the clinic is different than what the doctor is used to.”
PAP Application Process	“Getting the income documents from patients is the hardest, but knowing where the application is in the submission process is really tough.”
